# School closures help reduce the spread of COVID-19: A pre- and post-intervention analysis in Pakistan

**DOI:** 10.1371/journal.pgph.0000266

**Published:** 2022-04-20

**Authors:** Abdul Mueed, Razia Aliani, Mujahid Abdullah, Twangar Kazmi, Faisal Sultan, Adnan Khan

**Affiliations:** 1 Akhter Hameed Khan Resource Center, Islamabad, Pakistan; 2 Ministry of National Health Services, Regulation and Coordination, Islamabad, Pakistan; 3 Research and Development Solutions, Islamabad, Pakistan; Universite de Montreal, CANADA

## Abstract

Closing schools to control COVID-19 transmission has been globally debated, with concerns about children’s education and well-being, and also because of the varied effectiveness of the intervention in studies across the world. This paper aims to determine the effect of school closure policy on the incidence of COVID-19 in Pakistan. A Difference-in-Differences (DiD) analysis compared changes in COVID-19 incidence across cities that completely (Islamabad) and partially (Peshawar) closed schools during the second wave of COVID-19 in Pakistan. Effects of closing (November 2020) and reopening schools (February 2021) were assessed in Islamabad and Peshawar 10 and 20 days after policy implementation. In Islamabad, there was a greater decline in cases than in Peshawar when schools closed. After 10-days, the average reduction of daily COVID-19 incidence in Islamabad was lower by 89 cases (95% CI: -196, 18), due to complete school closure, with a relative reduction of 125 cases (95% CI: -191, -59) compared to Peshawar. Similarly, the relative increase in Islamabad after schools re-opened was 107 cases (95% CI: 46, 167) compared to Peshawar. After 20-days, the average daily COVID-19 incidence in both cities declined after school were closed (Islamabad: -81 [95% CI: -150, -13] versus Peshawar: -80 [95% CI: -148, -12]). COVID-19 incidence appeared to decline after schools reopened as well (Islamabad: -116 [95% CI: -230, -3] versus Peshawar: -30 [95% CI: -124, 63]). However, Peshawar’s decline is not statistically significant. These results control for changes in testing as well as a daily time trend. The magnitude and speed of reduction in cases with a complete school closure, and a similar but reverse trend of increasing cases upon reopening, suggests that closing schools reduces COVID-19 transmission in communities. However, there are learning-loss and well-being costs for children and their parents.

## Introduction

Since the beginning of the COVID-19 epidemic, societies have sought to control spread of the disease through Non-Pharmaceutical Interventions (NPI)—either barriers or by limiting contact between people. However, as the pandemic stretched on, initial mass lockdowns evolved into nuanced closures of commercial activity, public spaces public transport, educational institutions, workplaces and more. Over time, each of these were further refined. For example, within the education sector, attempts were made to understand which group of students or institutions could be focused upon for maximal impact on the epidemic while minimizing costs.

Any potential effectiveness of closing schools on reducing COVID-19 incidence must be balanced against the substantial costs of such closures [1]. These costs may be economic [2], in terms of learning losses [3] and even on social good. For example, in some developing countries children receive free lunch meals and prolonged school closures can lead to malnutrition, indirectly worsening learning outcomes [4]. While educators have sought to reduce learning losses through remote learning, such means are unaffordable for the poorest students, may unduly increase workload for teachers [5], and adversely affects children’s mental health [6]. These in turn further exacerbate the existing educational divides [7], particularly in developing countries like Pakistan where literacy is 57% [8] and 23 million or 44% of all school age children are out of school [9].

Not surprisingly then, the value of extended or repeated closure of schools has been questioned. In some contexts, their benefits may not outweigh the costs [10] and this has led to some countries to reopen their schools, albeit with precautions. For example, in the US, a “layered” system of face coverings/masks, social distancing and hygiene practices was recommended by the country’s Centers of Disease Control [11]. However, in countries such as Pakistan, where as much as 59% of all urban school attendance is in private schools [12], many of them low-cost, such precautions are not always possible. Since in the March to November 2020 period, school closures were considered the main policy option each time COVID-19 cases increased, this analysis was conducted to test whether the policy made scientific sense.

On November 26, 2020, schools all over Pakistan were ordered to close completely. However, the Khyber Pakhtunkhwa (KP) province allowed schools to open for one day a week. Attendance days were staggered across classes, and at least some children attended school on any working day. Schools eventually reopened all over Pakistan starting in late January 2021. Enforcement of school closures was universal and ensured by the district administration with military support. This varied approach to school closures by KP allowed for a natural experiment to compare the impact of partial (in Peshawar, KP) vs. full (in Islamabad) closure and reopening of schools on incident COVID-19 cases through a pre- and post-intervention analysis using a Difference-in-Differences (DiD) technique. DiD is a quasi-experimental approach that allows quantification of differential effect of a treatment or policy change on intervention group versus control group. The closing of educational institutions in November 2020 was the only NPI that was enacted on its own. Other NPIs, throughout 2020, were always enacted in “buckets”; for example, mask-wearing along with restrictions on the number of people at weddings and restrictions of public gatherings.

## Methodology

### Data

We used a DiD approach to compare pre- and post-school closure changes in daily incidence of COVID-19 cases in Islamabad (treatment group) with the corresponding change in Peshawar (control group). Dates for school closures and school reopening are considered interventions in our estimated model. Schools in Islamabad were closed completely, whereas in Peshawar, they were closed partially, that is, they were open for each class for one day per week, with attendance for each class staggered throughout each week. The directive to close all schools to control the spread of COVID-19 was enforced by district administrations with support from the military and were universal, “without exception.” [13] However, since education is a devolved governance subject in Pakistan, each federating unit’s government has the authority to choose the specific manner in which the school closure NPI was implemented; hence the partial closure of schools in Peshawar by the government of KP and the complete closure of schools in Islamabad. Nevertheless, the order to close schools was applicable to all pupils for all education institutes in each of the two cities, whether boarding schools or day schools–the latter of which form the vast majority of all schools in both cities.

Consequently, our findings speak to the difference in COVID-19 case reduction based on a complete closure versus a partial closure of schools. In other words, the findings of our estimation are applicable to the entire populations of Islamabad and Peshawar, irrespective of age, gender, type of schools (boarding or day) or pupils as disaggregation by demographics was not available in official COVID-19 statistics. See S1 Text for details on how the statistical analysis was conducted in STATA.

**Fig 1:**
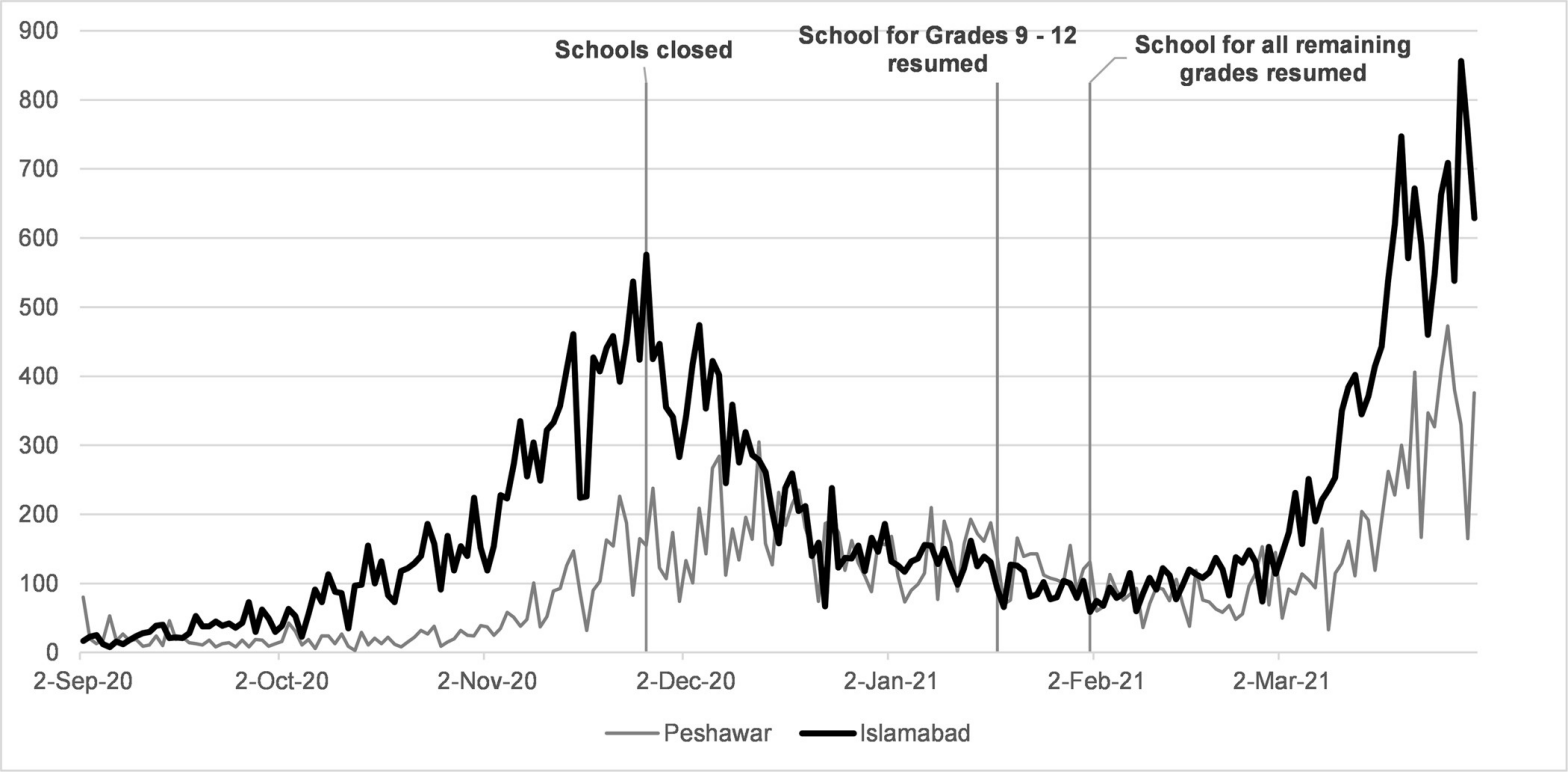
Daily new COVID -19 cases: Islamabad vs Peshawar (mid-September 2020 –End Feb 2021).

Data for this analysis is based on all daily COVID-19 tests (PCR and rapid antigen) and daily COVID-19 cases from the daily National Situational Reports of the National Emergency Operations Center (NEOC) aggregated by city level. Fig 1 plots the incidence of daily COVID-19 cases along with the chronology of NPIs from mid-September 2020 to the end of March 2021. Our city-level time-series data spans over a period of two months from November 06, 2020 to January 04, 2021 for school closures and January 12, 2021 to March 12, 2021 for the re-opening of schools.

Islamabad and Peshawar were chosen as treatment and control, respectively, because both cities share similar characteristics. A key condition for a DiD analysis is the presence of similarities between the control and treatment group. They are located close to each other in the north of Pakistan and have a comparable climate, and both also have frequent intra-city travel between each other. Both cities’ population demographics lend themselves to a comparison. Islamabad’s urban population is 1.01 million [14], whereas Peshawar’s urban population is 1.89 million [15]. The population density of Islamabad is 2,211 people per KM^2^ [16], whereas that of Peshawar is 1,849 people per KM^2^ [17].

**Fig 2:**
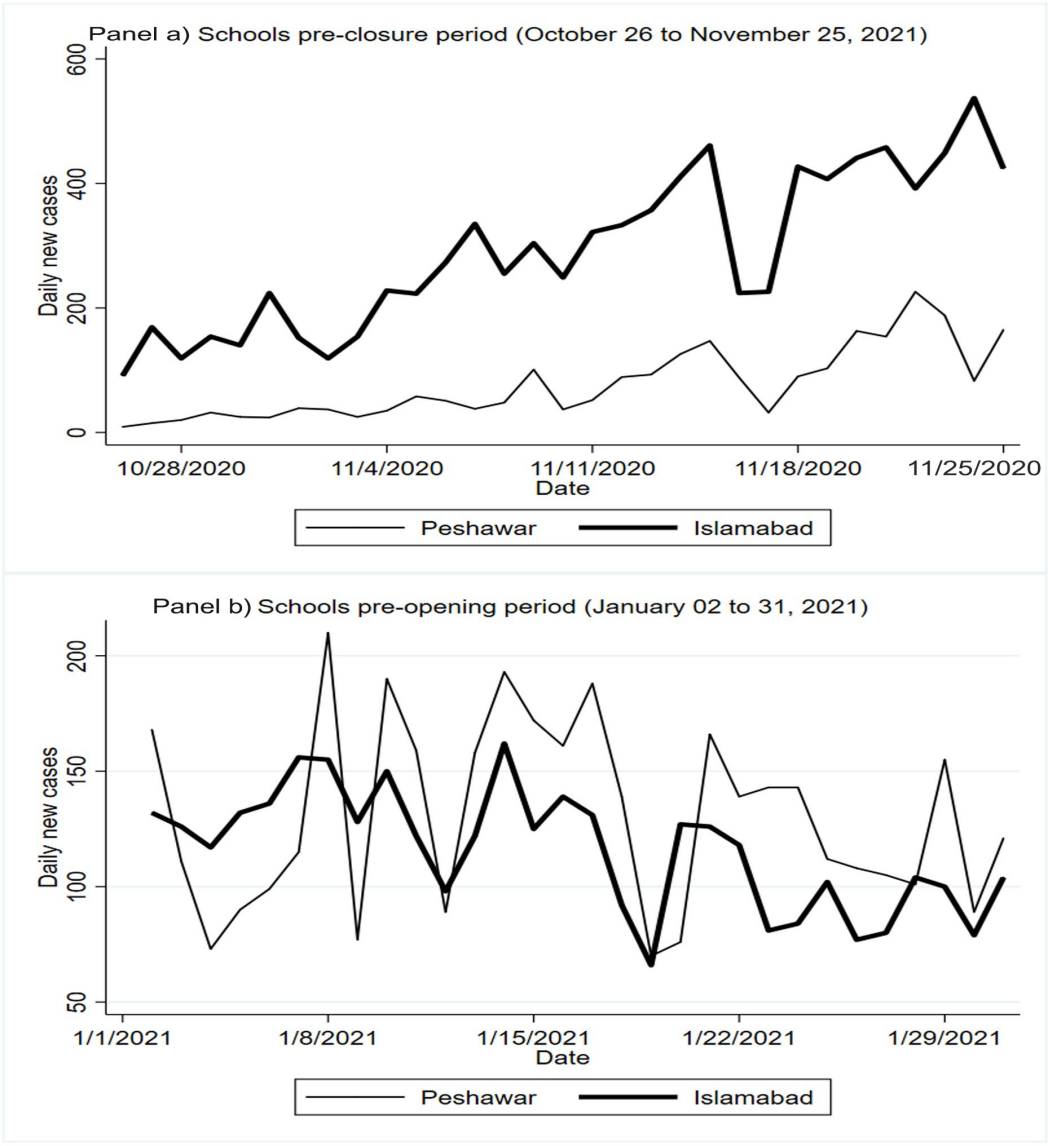
COVID-19 incidence in Islamabad and Peshawar pre-school closure and pre- school reopening.

More importantly, the trend of COVID-19 cases in each city, before the closure of schools and then later before the re-opening of schools, paralleled each other. A key assumption of a DiD regression is that the outcome, i.e., COVID-19 incidence, has a parallel trend in the control and intervention groups. Our analysis shows that Islamabad and Peshawar had a similar trend in daily new COVID-19 cases, both, one month before the schools were closed and also a month before they were subsequently re-opened (Fig 2).

Pre-intervention parallel trends were established using graphical observation, which has precedence in the context of DiD in literature [18], primarily because it appeared that quantitative measures to establish pre-intervention parallel trends are problematic/biased in their own right [19–21] thus an insufficient replacement for the logical assumption of a parallel trend, based key factors like demographics–as done above.

Additionally, since no other NPIs were contemporaneous with the school closure policy that could also affect COVID-19 transmission across our control and treatment groups, violation of DiD parallel trends assumption from contemporaneity is less likely.

Given the aforementioned parallel trend, comparable city characteristics and the government of KP’s differentiated approach to school closures, Islamabad and Peshawar serve as a viable treatment-control pair.

To account for a delayed effect of school closures on COVID-19 cases, we also took the intervention date 10 and also 20 days after the actual implementation of the school closure policy [22]. It was our assumption that the full effects of closing schools may not be realized until some time has passed after the intervention. Therefore, we considered December 6, 2020 and December 16, 2020 instead of November 26, 2020, as the intervention date for school closures in both cities.

To assess the effects of re-opening schools in both cities from a complete closure to a complete re-opening (for Islamabad), and from a partial closure to complete re-opening (for Peshawar). For the reopening of schools as well, we accounted for a 10 and 20-day delay by taking the pre-treatment period from January 12, 2021 to February 10, 2021, and the post-treatment period from February 11, 2021, rather than February 1, 2021, to March 12, 2021.

In order to check for serial/autocorrelation, we calculated all of our DiD estimates (that is for school closures and school reopening, each with 10-day and 20-day delays in Islamabad as well as Peshawar) using New-West standard errors. For each of these regressions, the residuals were assessed using ACF and PACF tests, at the appropriate lag, and were found to have been adjusted for all serial correlation at the identified lag structure. See S1 Text for details of all lags. We have also adjusted our model for a time trend. Due to the short total 60-day span of each pre- and post-intervention period (for school closures and for re-opening), we assumed no additional time-varying factors besides people’s mobility and daily new COVID-19 tests. We adjusted our final estimation for the quantum of testing, but not for mobility due to a lack of reliable data measuring the latter variable on a city level for Peshawar and Islamabad.

### Statistical analysis

We used the time-series data to estimate a difference-in-differences regression model, in which our dependent variable was number of COVID-19 cases in each group (*i*) per day (*j*). We adjusted our regression model for the variation in the quantum of daily testing and included a daily time trend term:

$$Y_{ij}=\alpha_{00}+\gamma_{01}T_{i}+\gamma_{10}t_{j}+\gamma_{11}\;T_{i}*t_{j}+\delta X_{ij}+\vartheta \;T_{ij}^{\prime }+\varepsilon_{ij}$$

Where *Y*_*ij*_ = daily number of COVID-19 cases (dependent variable)

$$T_{i}=Treatment\;variable=\left\{ \begin{array}{c} \;1\;if\;Islamabad\\ 0\;if\;Peshawar \end{array}\right.$$

$$t_{j}=Period\;variable=\left\{ \begin{array}{c} \;1\;for\;post-schools\;closure\;\\ 0\;for\;pre-schools\;closure\; \end{array}\right.$$

*γ*_01_ = coefficient measuring differences between Islamabad and Peshawar before intervention

*γ*_10_ *=* coefficient measuring shared differences in both cities across time

*γ*_11_ = coefficient measuring causal effect of school closure on number of cases in Islamabad

*T*_i_*t_*j*_ *=* The difference-in-differences term; interaction time between treatment and period variables (our focal independent variable)

*δ* = coefficient for daily testing (*X*_*ij*_)

*ϑ* = coefficient for daily time trend ($T_{ij}^{\prime }$)

*ε*_*ij*_ = unobservable factors

where the DID average treatment effects estimator (*γ*_11_) is given as:

$$\gamma_{11}=\{E[Y_{ij}|T_{1},t_{1}]-E[Y_{ij}|T_{0},t_{1}]\}-\{E[Y_{ij}|T_{1},t_{0}]-E[Y_{ij}|T_{0},t_{0}]\}$$

We performed our statistical analysis using STATA version 16. We adjusted our model for the quantum of daily testing across intervention and control cities. We also calculated COVID-19 incidences before and after 10 and 20 days of policy implementation (both when schools were closed and subsequently reopened) in Islamabad and Peshawar using OLS regressions. In every case, statistical significance of 5% (p<0.05) or less was considered with robust standard errors.

## Results

### Descriptive statistics

Descriptive statistics (Table 1) show that, on average, prior to school closures, there were 377 daily new COVID-19 cases in Islamabad and 118 in Peshawar, with a difference of 259 cases. Once schools closed, the daily average number of new cases decreased to 209 in Islamabad and increased to 168 in Peshawar, a difference of 41 incident cases daily.

**Table 1:** Descriptive statistics pre-and post–policy implementation across Intervention and control cities.

		Islamabad			Peshawar		
		N	Mean (SD)	Range	N	Mean (SD)	Range
				(min-max)			(min-max)
**School Closure**	**Daily new Cases**						
	Pre-Intervention	30	376.6 (88.4)	224–576	30	117.7 (56.4)	32–238
	Post Intervention	30	209 (88.6)	67–422	30	167.5 (57.6)	73–305
	**Daily new tests**						
	Pre-Intervention	30	6232.1 (120.2)	1917–8016	30	1023.9 (243.6)	378–1357
	Post Intervention	30	4771 (1133.2)	2560–6852	30	1623.6 (615.8)	521–2745
**School re-opening**	**Daily new Cases**						
	Pre-Intervention	30	98.2 (25.2)	59–162	30	114.9 (40.8)	36–193
	Post Intervention	30	159.2 (75.8)	74–384	30	92.4 (36.6)	33–179
	**Daily new tests**						
	Pre-Intervention	30	5563.7 (918.2)	3557–7102	30	1298.9 (215.2)	596–1783
	Post Intervention	30	4838.4 (1217.1)	3150–9240	30	1544.4 (126.3)	1402–1853

Similarly, on schools reopening in February 2021, the average number of daily new COVID-19 cases in Islamabad increased from 98 before school reopening to 159 cases after schools were re-opened. In Peshawar, the average daily new cases were 115 pre-reopening and decreased to 94 after schools were re-opened.

COVID-19 testing in Islamabad was nearly 6 times more than in Peshawar before schools were closed. However, after the closure of schools, testing in Islamabad decreased, but increased slightly in Peshawar. Before schools were re-opened, there remained no significant difference in testing across two cities. Once schools re-opened, testing significantly increased in Islamabad.

### Difference in difference estimates

#### 10-Days after NPI implementation

**Fig 3:**
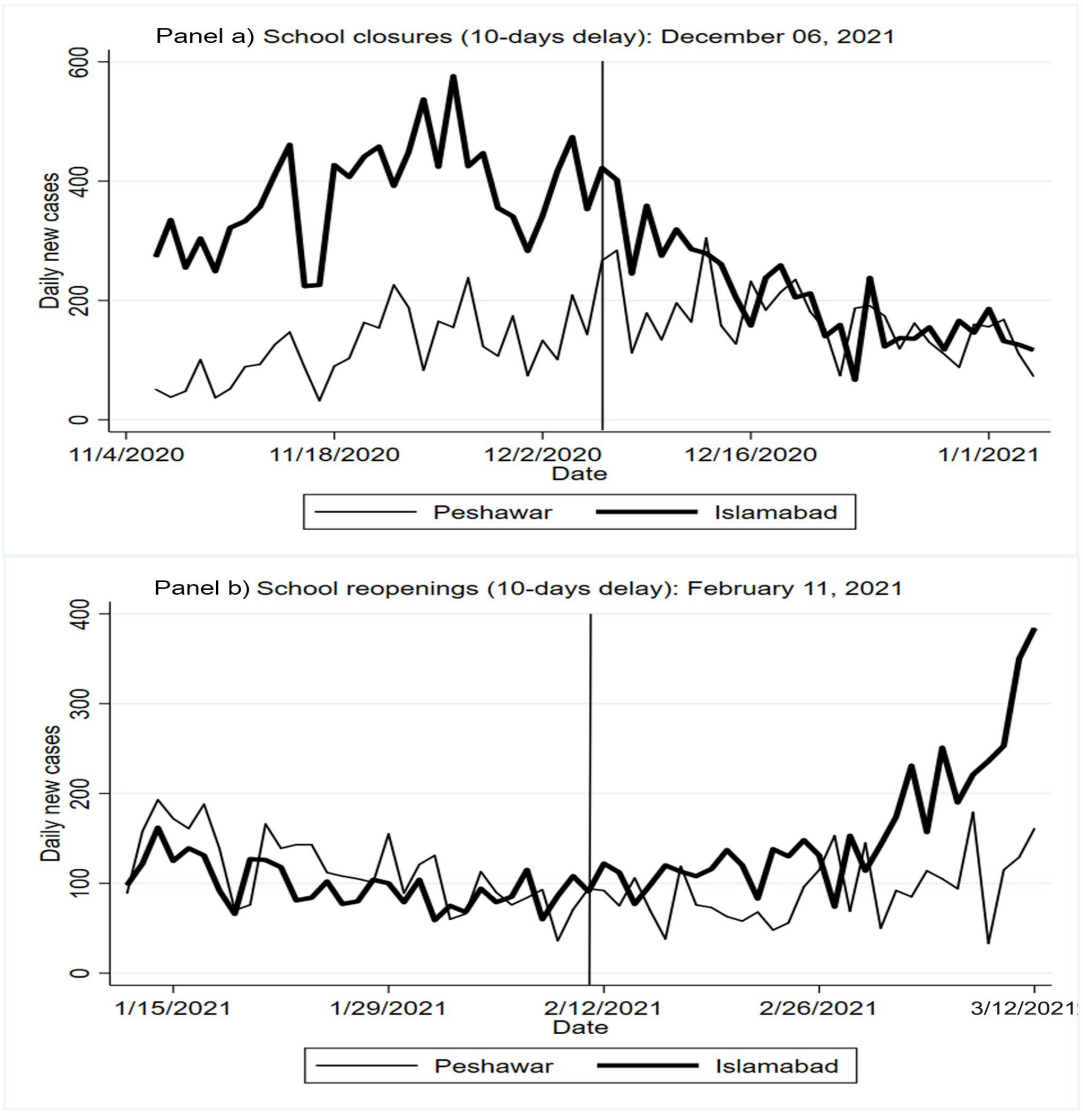
Daily COVID-19 cases in Islamabad and Peshawar pre- and post- school closure and school re-opening schools.

There was a statistically significant decline and subsequent statistically significant increase in COVID-19 incidence after a complete school closure versus a partial school closure and reopening, respectively (Table 2, Fig 3).

**Table 2:** Difference in COVID-19 incidence pre- and post- school closure and re-opening across Islamabad and Peshawar and between pre-and post- implementation in Islamabad and Peshawar 10 days after NPI implementation.

	10 Days Delay		
	Unadjusted β (95% CI), p-value	Adjusted β** (95% CI), p-value	R^2^
**School Closure** *	-217.4 (-295.0, -139.8), p<0.01	-124.8 (-190.2, -59.36), p<0.01	74%
Islamabad	-167.6 (-237.1, -98.05), p<0.01	-89.19 (-196.2, 17.8)	67%
Peshawar	49.83 (6.867, 92.80), p<0.05	27.69 (-56.90, 112.3)	26%
**School Reopening** *	83.53 (18.74, 148.3), p<0.05	106.6 (45.76, 167.4), p<0.01	37%
Islamabad	60.97 (0.4837, 121.4), p<0.01	1.156 (-69.14, 71.45)	48%
Peshawar	-22.57 (-51.62, 6.482), p<0.05	-9.79 (-60.14, 40.56)	41%

*Values are difference-in-differences showing change over time in COVID-19 incidence between Islamabad minus change over time in Peshawar.

** Adjusted for daily COVID-19 tests and with a daily time trend.

Ten days after schools were closed in late November 2020, Islamabad had a relative reduction of 125 daily COVID-19 cases (95% CI: -190, -59), compared to Peshawar. The decline in daily COVID-19 incidence in Islamabad in the post-intervention period, after considering a 10-day delay from when schools were closed, was 89 cases (95% CI: -196, 18) but this is not statistically significant at 95%. In Peshawar, however, there appeared to be an increase in COVID-19 incidence ten days after schools were partially closed; but this increase is not statistically significant.

Schools were reopened all over Pakistan starting in late January 2021. Classes for grades 9–12 were resumed from January 18, 2021 while those for grades 1–8 were resumed from February 1, 2021 [22]. Ten days after all the schools reopened from a complete closure in Islamabad, there was a relative increase of 107 COVID-19 cases (95% CI: 46, 167) per day in Islamabad, compared to Peshawar. The post-intervention increase in daily COVID-19 incidence in Islamabad, after considering a 10-day delay, was 1.2 cases (95% CI: -36.5, 38.8), however this is not statistically significant.

20- Days after NPI implementation. Twenty days after schools were completely closed, Islamabad had a relative average decrease of 132 daily COVID-19 cases (95% CI: -178, -86), compared to Peshawar. The decline in daily COVID-19 incidence in Islamabad was 81 cases (95% CI: -150, -13), 20 days post- school closure, compared to when schools were still open. In Peshawar, while COVID-19 cases continued to rise after partial school closures, they did fall significantly after 20 days, a decrease of 80 cases (95% CI: -148, -12) on average (Table 3).

**Table 3:** Difference in COVID-19 incidence pre- and post- school closure and re-opening across Islamabad and Peshawar and between pre-and post- implementation in Islamabad and Peshawar 20 days after NPI implementation.

	20 Days Delay		
	Unadjusted β (95% CI), p-value	Adjusted β** (95% CI), p-value	R^2^
**School Closure** *	-209.2 (-270.8, -147.6), p<0.01	-132.1 (-178.5, -85.6), p<0.01	76%
Islamabad	-215.7 (-267.6, -163.8), p<0.01	-81.4 (-149.5, -13.30), p<0.05	80%
Peshawar	-6.5 (-35.8, 22.8)	-80.1 (-148.3, -11.8), p<0.05	33%
**School Reopening** *	152.6 (-23.74, 328.9), p<0.1	154.1 (48.87, 259.3), p<0.01	66%
Islamabad	198.4 (44.07, 352.7), p<0.05	-116.3 (-229.9, -2.742), p<0.05	78%
Peshawar	45.8 (-15.44, 107.0)	-30.49 (-124.1, 63.12)	30%

*Values are difference-in-differences showing change over time in COVID-19 incidence between Islamabad minus change over time in Peshawar.

** Adjusted for daily COVID-19 tests and with a daily time trend.

Twenty days after all schools reopened from complete closure, there was a relative increase of 154 COVID-19 cases (95% CI: 49, 259) per day in Islamabad compared to Peshawar. The reduction in daily COVID-19 incidence in Islamabad was 116 cases (95% CI: -230, -3), 20 days post-school re-opening. In Peshawar, however, there was no statistically significant difference in COVID-19 incidence 20 days after schools were fully re-opened. For Islamabad, the 20-day delay results for schools’ re-opening suggest that there was some role played by seasonality, or other confounders, in the upsurge on COVID-19 cases after schools were re-opened in Islamabad.

## Discussion

We showed, using DiD from COVID-19 data for Islamabad and Peshawar, that school closures appear to lower new COVID-19 cases in both cities. Additionally, the reduction appears more pronounced in Islamabad, where schools were completely closed, compared to Peshawar where schools remained open one day a week. In Peshawar, the reduction in COVID-19 cases was more modest and was delayed by 10 days after the intervention. This effect is further validated by the observation of a reverse trend, i.e. a rise in COVID-19 cases upon re-opening of schools.

These effects should be viewed in the context of both cities’ population demographics. Islamabad is the more densely populated city, with people living closer to each other and coming into more frequent contact as a result. However, the overall population in Peshawar is larger than that of Islamabad. More importantly, testing in Peshawar was, proportionally, significantly lower than in Islamabad throughout the period of observation for this study (see Table 1), meaning there could be issues with capturing the true burden of the disease.

Nevertheless, our findings are consistent with published research on the effects of school closures around the world. Much of this literature finds that, in general, closing schools reduced cases, hospitalizations and deaths [23–26]. There is also evidence for infection risk being possibly higher between same-age contacts, particularly among children aged 0 to 14 years [27].

This is an important consideration in a country like Pakistan, where many schools, even private ones, are small and crowd as many as 100–150 children in 250–300 square meter space for up to 5–6 hours. This is in addition to many children commuting to and from school in a cramped, privately-run pick-and-drop van, with as many as 15 children per van. All of these factors possibly make schools in Pakistan a so-called super spreader location.

However, evidence from around the world also suggests that while closing both schools plus higher education institutions reduces the spread of COVID-19 [28], closing higher education institutions may be more effective for controlling cases than closing schools [29]. Additionally, it is better to act quickly in closing educational institutes to effectively reducing transmission [30, 31]. Moreover, reopening schools could lead to a spike in cases and the spread of the infection [32, 33].

In contrast, there is considerably less evidence to support the notion that there is no effect [10, 34–36] of closing schools or that there are ambiguous/mixed effects of school closures on cases and deaths [37–40]. A number of factors can account for these diminished outcomes, including the fact that it is difficult to separate the effects of closing schools from those of other NPIs.

There is also speculation that the frequent previous exposure of children to upper respiratory tract infections, including coronaviruses, may have conferred them with protective antibodies [41]. There is no concrete, recent evidence to support this in Pakistan, but it is still a sufficiently generalizable medical phenomenon. In the case of Pakistan, it is also useful to note that some schools, especially private ones in affluent locales, can provide more space to children for social distancing, or might provide barriers in classrooms.

Overall, it is likely that the experience with school closures will be mixed and dependent on the local context. Factors such as the crowding of children in school or during transport and prior exposures to pathogens may play a role in eventual outcomes.

### Limitations

There are a number of limitations to our analysis, that relate to the methodology and stem from the data that was available, or rather not available.

One key variable on which data was not available is secondary COVID-19 infections, i.e., people who are infected from someone who is already infected. The lack of this crucial variable did not allow us to model the dynamics of the transmission of COVID-19 in Pakistan. At present, there is also no example of this for Pakistan in any published research. As such, our analysis is unable to account for disease dynamics in Pakistan, and should thus be read as such.

We also do not have data for COVID-19 infections that is disaggregated by age, gender or schools. The official statistics we have access to only aggregate data on the national and sub-national level, as well as for major cities like Peshawar and Islamabad. With regards to mobility, there is no data that records intra-city mobility in Pakistan. For inter-city mobility, Google’s Community Mobility Reports, though instructive, were nevertheless not found to be suitable, based on the data’s collection methodology, due to their very limited representation of the population of both Peshawar and Islamabad.

An additional limitation of the data available to us was the underlying nature of the data for our dependent variable, daily new COVID-19 cases. The mean and variance of this variable over our period of observation are not equal. This precluded the possibility of using, for example, a Poisson regression. A negative binomial (NB) regression was also ruled out due to the lack of data on disease dynamics for Pakistan, which would be necessary for such an analysis using NB [42]. Moreover, in the case of both methods, a key assumption is for two observations to be independent of each other. With regards to daily COVID-19 infections, it is our contention that such independence is theoretically not possible, as COVID-19 is a communicable infectious disease.

Our choice of DiD was also motivated by the relative ease in communicating its findings to policymakers, as our goal was to try and shed light of COVID-19’s responsiveness to policy changes.

Nevertheless, we acknowledge that a pre/post intervention analysis in itself has limitations. For example, it is not effective at determining the effects of more than one intervention at a time. This is referred to as the “history threat” to the internal validity of a pre/post intervention analysis, whereby an event other than the treatment itself may accounts for the observed change and potentially confound assessment of competing interventions [43]. However, in Pakistan, as elsewhere, multiple NPIs were implemented simultaneously. Since other NPIs were implemented to the same degree in Islamabad and Peshawar, our DiD analysis would be appropriate to assess school closures and school re-opening.

In addition to the above, it is possible that COVID-19 cases in Islamabad and Peshawar may have changed as a result of something, possibly unobserved, that happened *before* schools were closed, such as the possibility that rising cases may have changed public behavior, e.g., to be more careful, thus helping lower cases. This is referred to as the “testing threat” to the internal validity of a pre/post intervention analysis, whereby the dependent variable might be changing as a result of something that happened before the intervention. This is difficult to address but we are aware of no data to suggest such a confounder [43].

Additionally, we acknowledge that in the case of a pandemic like COVID-19, it is possible that cases from one city might affect a nearby city. Islamabad and Peshawar are only a 2-hour drive apart from each other. However, there is no data that can be cited to account for the magnitude of any potential contamination in our model due to intra-city travel between our control and treatment groups. Additionally, such bilateral travel may well *decrease* the difference in the number of COVID-19 cases between the two cities, rather than increasing it as our results indicate.

Moreover, given our data limitations, and thus our need for using DiD, our control and treatment groups needed to be sufficiently similar. Out of all the major cities in Pakistan, no two cities are as comparable as Islamabad and Peshawar, much less comparable cities that also used differential approaches to the same NPI.

Furthermore, there is discourse around Pakistan’s case detection rate being lower than the actual cases in the community, due to low testing levels [44], or perhaps even late reporting. It is beyond the scope of this study to ascertain the magnitude of this effect. However, even if it is true, it is unlikely to affect the analysis as the testing strategy and practices remained consistent across the country during the study periods. Moreover, while taking a 1-month period for a pre/post intervention analysis does not introduce too many time-varying factors, it is nevertheless a long enough period to smooth over any fluctuations caused due to late reported infections.

Given the methodological and data challenges in our analysis, documenting the dynamics of COVID-19 in Pakistan as well as ascertaining more ideal methodologies to assess the effects of policy changes are both topics further research.

Despite all the limitations stated above, we maintain that our findings are still sufficiently indicative of disease response to pandemic policy in the context of Pakistan, given the dearth of literature in this very specific regard.

## Conclusions

The changing nature of the virus (with differences in transmissibility and virulence of the newer mutants and highly varied nature, customs and resources of the societies that it has infected), along with widely differing social contexts across the world have made for an incredibly complex and changing policy landscape where any NPI, such school closures, may be understood. Our findings suggest that school closures curtail transmission of COVID-19, likely in less affluent communities, where the majority of schools and students cannot socially distance during their commutes or in the classroom, or to allow barrier protection such as shields. These same factors also raise the social costs of school closures for a developing country such as Pakistan. Given our data and methodological limitations, further research into this matter is still important.

## Supporting information

S1 Table Difference-in-differences estimates: School closures with 10-days delay. (PDF)

S2 Table Regression estimates with 10-days delay–Islamabad pre- and post-closure. (PDF)

S3 Table Regression estimates with 10-days delay–Peshawar pre- and post-closure. (PDF)

S4 Table Difference-in-differences estimates: School re-openings with 10-days delay. (PDF)

S5 Table Regression estimates for Islamabad–Re-openings with 10-days delay. (PDF)

S6 Table Regression estimates for Peshawar–Re-openings with 10-days delay. (PDF)

S7 Table Difference-in-differences estimates: School closures with 20-days delay. (PDF)

S8 Table Regression estimates with 20-days delay–Islamabad pre- and post-closure. (PDF)

S9 Table Regression estimates with 20-days delay–Peshawar pre- and post-closure. (PDF)

S10 Table Difference-in-differences estimates: School re-openings with 20-days delay. (PDF)

S11 Table Regression estimates for Islamabad–Re-openings with 20-days delay. (PDF)

S12 Table Regression estimates for Peshawar–Re-openings with 20-days delay. (PDF)

S1 Text Methodology steps. (PDF)

S1 Data Full dataset (cleaned). (XLSX)
