# Supplementary material for: School closures help reduce the spread of COVID-19: A pre- and post-intervention analysis in Pakistan
Source: PLOS Glob Public Health. 2022 Apr 20;2(4):e0000266. doi: 10.1371/journal.pgph.0000266 (PMC10021268; doi:10.1371/journal.pgph.0000266)
Supplement: S1 Table — (PDF) [file pgph.0000266.s001.pdf]

S1 Table: Difference-in-Differences Estimates: School closures with 10-days delay

VARIABLES	(1)<br>Daily new cases	(2)<br>Controlled for daily tests and time trend
Treatment variable =1 if Islamabad	258.9***	24.88
	(205.0, 312.8)	(-56.97, 106.7)
Period variable =1 if Post-closure	49.83***	31.05
	(13.87, 85.80)	(-34.15, 96.24)
Diff-in-diff (*IslamabadxPost-closure*)	-217.4***	-124.8***
	(-295.0, -139.8)	(-190.2, -59.36)
Daily new tests		0.0450***
		(0.030, 0.0598)
Time		-0.2720
		(-2.168, 1.624)
Constant	117.7***	75.91***
	(88.99, 146.4)	(25.98, 125.8)
Observations	120	120
R-squared	0.639	0.745

Newey-West standard errors used, CI in parentheses

*** p<0.01, ** p<0.05, * p<0.1
