# Supplementary material for: School closures help reduce the spread of COVID-19: A pre- and post-intervention analysis in Pakistan
Source: PLOS Glob Public Health. 2022 Apr 20;2(4):e0000266. doi: 10.1371/journal.pgph.0000266 (PMC10021268; doi:10.1371/journal.pgph.0000266)
Supplement: S2 Table — (PDF) [file pgph.0000266.s002.pdf]

S2 Table: Regression estimates with 10-days delay – Islamabad pre- and post-closure

VARIABLES	(1)<br>Daily new cases	(2)<br>Controlled for daily tests and time trend
Period variable =1 if Post-closure	-167.6***	-89.19
	(-237.1, -98.05)	(-196.2, 17.8)
Daily new tests		0.045***
		(0.028, 0.0618)
Time		-0.4203
		(-3.531, 2.691)
Constant	376.6***	102.6
	(330.4, 422.7)	(-25.04, 230.2)
Observations	60	60
R-squared	0.481	0.672

Newey-West standard errors used, CI in parentheses

*** p<0.01, ** p<0.05, * p<0.1
