# Supplementary material for: School closures help reduce the spread of COVID-19: A pre- and post-intervention analysis in Pakistan
Source: PLOS Glob Public Health. 2022 Apr 20;2(4):e0000266. doi: 10.1371/journal.pgph.0000266 (PMC10021268; doi:10.1371/journal.pgph.0000266)
Supplement: S3 Table — (PDF) [file pgph.0000266.s003.pdf]

S3 Table: Regression estimates with 10-days delay – Peshawar pre- and post-closure

VARIABLES	(1)<br>Daily new cases	(2)<br>Controlled for daily tests and time trend
Period variable =1 if Post-closure	49.83**	27.69
	(6.867, 92.80)	(-56.90, 112.3)
Daily new tests		0.0423**
		(0.0035, 0.0811)
Time		-0.107
		(-2.654, 2.44)
Constant	117.7***	76.07*
	(83.14, 152.2)	(-0.2259, 152.4)
Observations	60	60
R-squared	0.165	0.265

Newey-West standard errors used, CI in parentheses

*** p<0.01, ** p<0.05, * p<0.1
