# Supplementary material for: School closures help reduce the spread of COVID-19: A pre- and post-intervention analysis in Pakistan
Source: PLOS Glob Public Health. 2022 Apr 20;2(4):e0000266. doi: 10.1371/journal.pgph.0000266 (PMC10021268; doi:10.1371/journal.pgph.0000266)
Supplement: S4 Table — (PDF) [file pgph.0000266.s004.pdf]

S1 Table 4: Difference-in-Differences Estimates: School re-openings with 10-days delay

VARIABLES	(1)<br>Daily new cases	(2)<br>Controlled for daily tests and time trend
Treatment variable =1 if Islamabad	-16.73	-118.1***
	(-45.35, 11.88)	(-187.1, -49.1)
Period variable =1 if Post-opening	-22.57	-52.8
	(-51.31, 6.176)	(-122.3, 16.74)
Diff-in-diff (*IslamabadxPost-opening*)	83.53**	106.6***
	(18.74, 148.3)	(45.76, 167.4)
Daily new tests		0.0238***
		(0.001, 0.038)
Time		0.8131
		(-1.141, 2.768)
Constant	114.9***	71.46***
	(90.44, 139.4)	(24.61, 118.3)
Observations	120	120
R-squared	0.232	0.366

Newey-West standard errors used, CI in parentheses

*** $p<0.01$, ** $p<0.05$, * $p<0.1$
