# Supplementary material for: School closures help reduce the spread of COVID-19: A pre- and post-intervention analysis in Pakistan
Source: PLOS Glob Public Health. 2022 Apr 20;2(4):e0000266. doi: 10.1371/journal.pgph.0000266 (PMC10021268; doi:10.1371/journal.pgph.0000266)
Supplement: S5 Table — (PDF) [file pgph.0000266.s005.pdf]

S5 Table: Regression estimates for Islamabad – Re-openings with 10-days delay

VARIABLES	(1)<br>Daily new cases	(2)<br>Controlled for daily tests and time trend
Period variable =1 if Post-opening	60.97***	1.156
	(0.4837, 121.4)	(-69.14, 71.45)
Daily new tests		0.0208***
		(0.0097, 0.032)
Time		2.497*
		(-0.4303, 5.425)
Constant	98.2***	-56.38
	(82.75, 113.6)	(-155.0, 42.27)
Observations	60	60
R-squared	0.231	0.486

Newey-West standard errors used, CI in parentheses

*** p<0.01, ** p<0.05, * p<0.1
