# Supplementary material for: School closures help reduce the spread of COVID-19: A pre- and post-intervention analysis in Pakistan
Source: PLOS Glob Public Health. 2022 Apr 20;2(4):e0000266. doi: 10.1371/journal.pgph.0000266 (PMC10021268; doi:10.1371/journal.pgph.0000266)
Supplement: S6 Table — (PDF) [file pgph.0000266.s006.pdf]

S6 Table: Regression estimates for Peshawar – Re-openings with 10-days delay

VARIABLES	(1)<br>Daily new cases	(2)<br>Controlled for daily tests and time trend
Period variable =1 if Post-opening	-22.57**	-9.79
	(-51.62, 6.482)	(-60.14, 40.56)
Daily new tests		0.133***
		(0.771, 0.189)
Time		-1.52**
		(-2.706, -0.3244)
Constant	114.9***	-34.42
	(90.18, 139.7)	(-119.7, 50.89)
Observations	60	60
R-squared	0.081	0.410

Newey-West standard errors used, CI in parentheses

*** p<0.01, ** p<0.05, * p<0.1
