# Supplementary material for: School closures help reduce the spread of COVID-19: A pre- and post-intervention analysis in Pakistan
Source: PLOS Glob Public Health. 2022 Apr 20;2(4):e0000266. doi: 10.1371/journal.pgph.0000266 (PMC10021268; doi:10.1371/journal.pgph.0000266)
Supplement: S7 Table — (PDF) [file pgph.0000266.s007.pdf]

S7 Table: Difference-in-Differences Estimates: School closures with 20-days delay

VARIABLES	(1)<br>Daily new cases	(2)<br>Controlled for daily tests and time trend
Treatment variable =1 if Islamabad	212.5***	29.88
	(158.3, 266.7)	(-38.54, 98.3)
Period variable =1 if Post-closure	-6.5	2.834
	(-40.51, 27.51)	(-52.53, 58.19)
Diff-in-diff (*IslamabadxPost-closure*)	-209.2***	-132.1***
	(-270.8, -147.6)	(-178.5, -85.6)
Daily new tests		0.0369***
		(0.0233, 0.0505)
Time		-0.9128
		(-2.433, 0.6073)
Constant	155.8***	128.1***
	(129.2, 182.5)	(88.88, 167.2)
Observations	120	120
R-squared	0.679	0.760

Newey-West standard errors used, CI in parentheses

*** p<0.01, ** p<0.05, * p<0.1
