# Supplementary material for: School closures help reduce the spread of COVID-19: A pre- and post-intervention analysis in Pakistan
Source: PLOS Glob Public Health. 2022 Apr 20;2(4):e0000266. doi: 10.1371/journal.pgph.0000266 (PMC10021268; doi:10.1371/journal.pgph.0000266)
Supplement: S8 Table — (PDF) [file pgph.0000266.s008.pdf]

S8 Table: Regression estimates with 20-days delay – Islamabad pre- and post-closure

VARIABLES	(1)<br>Daily new cases	(2)<br>Controlled for daily tests and time trend
Period variable =1 if Post-closure	-215.7***	-81.4**
	(-267.6, -163.8)	(-149.5, -13.30)
Daily new tests		0.0304***
		(0.0144, 0.0463)
Time		-2.855***
		(-4.827, -0.8821)
Constant	368.4***	227.6***
	(320.7, 416.1)	(138.7, 316.5)
Observations	60	60
R-squared	0.693	0.803

Newey-West standard errors used, CI in parentheses

*** p<0.01, ** p<0.05, * p<0.1
