# Supplementary material for: School closures help reduce the spread of COVID-19: A pre- and post-intervention analysis in Pakistan
Source: PLOS Glob Public Health. 2022 Apr 20;2(4):e0000266. doi: 10.1371/journal.pgph.0000266 (PMC10021268; doi:10.1371/journal.pgph.0000266)
Supplement: S9 Table — (PDF) [file pgph.0000266.s009.pdf]

S9 Table: Regression estimates with 20-days delay – Peshawar pre- and post-closure

VARIABLES	(1)<br>Daily new cases	(2)<br>Controlled for daily tests and time trend
Period variable =1 if Post-closure	-6.5	-80.06**
	(-35.83, 22.83)	(-148.3, -11.81)
Daily new tests		0.0678***
		(0.0343, 0.0895)
Time		1.346
		(-0.2986, 2.99)
Constant	155.8***	57.9**
	(132.5, 179.2)	(6.021, 109.8)
Observations	60	60
R-squared	0.003	0.328

Newey-West standard errors used, CI in parentheses
*** p<0.01, ** p<0.05, * p<0.1
