# Supplementary material for: School closures help reduce the spread of COVID-19: A pre- and post-intervention analysis in Pakistan
Source: PLOS Glob Public Health. 2022 Apr 20;2(4):e0000266. doi: 10.1371/journal.pgph.0000266 (PMC10021268; doi:10.1371/journal.pgph.0000266)
Supplement: S10 Table — (PDF) [file pgph.0000266.s010.pdf]

S10 Table: Difference-in-Differences Estimates: School re-openings with 20-days delay

VARIABLES	(1)<br>Daily new cases	(2)<br>Controlled for daily tests and time trend
Treatment variable =1 if Islamabad	0.3333	-180.2**
	(-21.25, 21.91)	(-317.1, -43.23)
Period variable =1 if Post-opening	45.80	-151.2**
	(-16.47, 108.1)	(-278.7, -23.8)
Diff-in-diff (*IslamabadxPost-opening*)	152.6*	154.1***
	(-23.74, 328.9)	(48.87, 259.3)
Daily new tests		0.0471***
		(0.0156, 0.0787)
Time		6.118***
		(2.457, 9.78)
Constant	94.73***	-63.58
	(76.04, 113.4)	(-171.6, 44.46)
Observations	120	120
R-squared	0.388	0.662

Newey-West standard errors used, CI in parentheses

*** p<0.01, ** p<0.05, * p<0.1
