# Supplementary material for: School closures help reduce the spread of COVID-19: A pre- and post-intervention analysis in Pakistan
Source: PLOS Glob Public Health. 2022 Apr 20;2(4):e0000266. doi: 10.1371/journal.pgph.0000266 (PMC10021268; doi:10.1371/journal.pgph.0000266)
Supplement: S11 Table — (PDF) [file pgph.0000266.s011.pdf]

S11 Table: Regression estimates for Islamabad – Re-openings with 20-days delay

VARIABLES	(1)<br>Daily new cases	(2)<br>Controlled for daily tests and time trend
Period variable =1 if Post-opening	198.4**	-116.3**
	(44.07, 352.7)	(-229.9, -2.742)
Daily new tests		0.0476***
		(0.0189, 0.0762)
Time		10.09***
		(5.577, 14.6)
Constant	95.07***	-307.46***
	(86.89, 105.2)	(-498.7, -116.2)
Observations	60	60
R-squared	0.365	0.777

Newey-West standard errors used, CI in parentheses

*** $p<0.01$, ** $p<0.05$, * $p<0.1$
