# Supplementary material for: School closures help reduce the spread of COVID-19: A pre- and post-intervention analysis in Pakistan
Source: PLOS Glob Public Health. 2022 Apr 20;2(4):e0000266. doi: 10.1371/journal.pgph.0000266 (PMC10021268; doi:10.1371/journal.pgph.0000266)
Supplement: S12 Table — (PDF) [file pgph.0000266.s012.pdf]

S12 Table: Regression estimates for Peshawar – Re-openings with 20-days delay

VARIABLES	(1)<br>Daily new cases	(2)<br>Controlled for daily tests and time trend
Period variable =1 if Post-opening	45.8	-30.49
	(-15.44, 107.0)	(-124.1, 63.12)
Daily new tests		0.1428**
		(0.0086, 0.2769)
Time		1.181
		(-2.815, 5.178)
Constant	94.73***	-115.8
	(76.47, 113.0)	(-290.5, 58.87)
Observations	60	60
R-squared	0.119	0.303

Newey-West standard errors used, CI in parentheses

*** p<0.01, ** p<0.05, * p<0.1
