# Supplementary material for: School closures help reduce the spread of COVID-19: A pre- and post-intervention analysis in Pakistan
Source: PLOS Glob Public Health. 2022 Apr 20;2(4):e0000266. doi: 10.1371/journal.pgph.0000266 (PMC10021268; doi:10.1371/journal.pgph.0000266)
Supplement: S1 Text — (PDF) [file pgph.0000266.s013.pdf]

S1 Text: Methodology steps

1. We took 60-day periods for each of our four average treatment effect analyses (10- and 20-days school closure and schools reopening).
2. We estimated our DiD estimators and kept intervention dates 10-days and 20-days after original dates of school closures (November 26, 2020) and schools reopening (February 1, 2021). We took the following steps in each of our four analyses:
   i. Labeled and converted data to categoric and specified the data as time series
   ii. Description of summary statistics
   iii. Data visualization for pre-intervention trends
   iv. To reduce bias and imbalance between treatment and control groups, the models were adjusted for daily tests and daily time trend.
   v. Final DiD models took daily COVID-19 cases as dependent variables, and interactions of city dummies and period dummies, daily new tests and daily time trends as independent variables.
   vi. Newey-West standard errors were used, which can correct for heteroskedasticity and serial/auto-correlation. In OLS regressions with Newey-West standard errors, correct lag structures were specified for unbiased estimates.
   vii. The serial/auto-correlation was then assessed through AC and PAC tests, which indicated that with correct lag structures, it was adjusted.
3. We then ran city-wise OLS regressions with Newey-West standard errors for each of our four intervention periods comparing pre- and post-intervention averages in daily cases in each district. The independent variables were period dummies, daily tests and daily time trends.
4. For school closures, we also tried to test the average treatment effects on multiple periods using the same dates of pre-intervention period (November 6, 2020 to December 5, 2020) but post-intervention dates differed, starting from 10-days post-intervention (December 6, 2020) and 20-days post-intervention (December 16, 2020) respectively. However, we do not use these results in our paper.
